# Randomized clinical trial of an enhanced recovery after surgery programme *versus* conventional care in laparoscopic Roux‐en‐Y gastric bypass surgery

**DOI:** 10.1002/bjs5.50143

**Published:** 2019-03-18

**Authors:** N. Geubbels, I. Evren, Y. I. Z. Acherman, S. C. Bruin, A. W. J. M. van de Laar, M. B. Hoen, L. Maurits de Brauw

**Affiliations:** ^1^ Department of Metabolic and Bariatric Surgery Slotervaart Medical Centre Amsterdam the Netherlands; ^2^ Department of Anaesthesiology Slotervaart Medical Centre Amsterdam the Netherlands

## Abstract

**Background:**

Enhanced recovery after surgery (ERAS) programmes have led to a decreased duration of hospital stay in several surgical fields, but have not been fully tested in patients undergoing laparoscopic Roux‐en‐Y gastric bypass (LRYGB) for obesity. This study aimed to investigate an ERAS programme *versus* standard care in these patients.

**Methods:**

Between January 2013 and July 2014, patients undergoing LRYGB were randomized to ERAS or conventional care. The primary outcome was functional hospital stay, defined as the time between end of surgery and when predefined discharge criteria (pain adequately controlled, fever and postoperative nausea and vomiting (PONV) absent, full liquid diet tolerated, mobilized and feeling fit for discharge) were met. Secondary outcomes were total length of hospital stay, 30‐day complication and mortality rates, duration of surgery, time spent on the recovery ward and health‐related quality of life.

**Results:**

A total 220 patients were randomized to ERAS (110 patients) or conventional (110) care. Patients in the ERAS group had shorter functional hospital stay (17·4 *versus* 20·5 h; *P* < 0·001), quicker pain control, tolerated liquid diet earlier, had earlier control of PONV, mobilized sooner and were comfortable with discharge sooner than those receiving conventional care. Total length of hospital stay, duration of surgery, time spent on the recovery ward, health‐related quality of life, complication and readmission rates did not differ between the study groups. There were no deaths.

**Conclusion:**

Patients under ERAS care recovered faster after LRYGB surgery than those receiving conventional care, with no increase in readmission and postoperative morbidity rates. Registration number: NTR3853 (http://www.trialregister.nl/).

## Introduction

Bariatric surgery is the most effective way to achieve sustainable weight loss and has a profound effect on obesity‐related co‐morbidities^1^, ^2^. The laparoscopic Roux‐en‐Y gastric bypass (LRYGB) procedure is one of the most widely used bariatric operations^3^. Mortality and short‐term adverse event rates after LRYGB have decreased significantly, from about 2 and 30 per cent respectively before the millennium^4^, ^5^ to around 0·2 and 4 per cent respectively in more recent studies^6^, ^7^, ^8^. Increasing patient volume is associated with this decrease in adverse events^9^, ^10^, ^11^. Enhanced recovery after surgery (ERAS) programmes are now used widely. They are designed to decrease the impact of the cascade of events caused by surgery^12^, including the metabolic and immune responses to surgery^13^, ^14^. ERAS programmes include preoperative counselling, a standardized protocol in anaesthetic management, standardized use of postoperative analgesics and antiemetics, restriction of tubes and catheters, early mobilization and early oral feeding^15^, ^16^.

ERAS programmes have proven particularly successful in colorectal surgery, where they have led to a decreased length of stay (LOS) and a beneficial effect on in‐hospital resources (reduction in surgical and transfer times)^17^. A fast‐track programme for patients having bariatric surgery at the authors' centre was introduced in 2011, and is thought to be partly responsible for a decrease in LOS from 3 days to 1 day^18^, although it is acknowledged that this reduction might also be attributable to increased expertise of the bariatric team. The only RCT^19^ performed in a bariatric population involved patients undergoing sleeve gastrectomy, and showed a decrease in LOS of 1 day, compared with 2 days in the conventional care group. The aim of the present study was to examine the contribution of an ERAS programme in patients undergoing LRYGB.

## Methods

This RCT was reviewed and approved by the medical ethics committee of the Slotervaart Medical Centre. The study protocol was registered in the Dutch Trial Register (trial number NTR3853). Patients were eligible for inclusion if they were aged 18–65 years and their BMI was 40 kg/m^2^ or above or 35 kg/m^2^ or more plus one or more of the obesity‐related co‐morbidities: hypertension, type 2 diabetes mellitus (T2DM), obstructive sleep apnoea, hypercholesterolaemia and osteoarticular disease. Exclusion criteria were patients with T2DM requiring insulin, residence more than 1 h by car from the hospital, ASA fitness grade more than III, requiring another surgical intervention besides the LRYGB in the same session, undergoing a revisional bariatric procedure, insufficient knowledge of the Dutch language and participation in any other (therapeutic) study that might influence the primary or secondary outcomes. Written informed consent was obtained from all the patients who agreed to participate in the study.

Patients were stratified between the surgeons to prevent any differences in outcome caused by surgeon‐related factors. With the help of an internet‐based randomization programme (https://www.graphpad.com/quickcalcs), patients were randomized into four groups (representing the four surgeons) between ERAS and conventional care. Randomization was 1 : 1. The allocation sequence was generated as a whole. Surgeons received their own basket with 60 concealed opaque envelopes and randomly picked an envelope from the basket at the outpatient clinic before the operation, after they had finished screening the bariatric surgery candidates. To avoid contamination, patients randomized to the ERAS group were scheduled on uneven week numbers and those in the conventional group on even week numbers.

All data were analysed on an intention‐to‐treat principle. This was an open‐label study; caregivers, patients, data collectors and analysts were not blinded.

### Study procedures

Patients in the intervention group received perioperative care according to an ERAS protocol, subdivided into preoperative, perioperative and postoperative sections (*Table* 1), following most of the 2016 recommendations regarding ERAS in bariatric surgery as suggested by Thorell and colleagues^20^. Patients in the conventional care group received care as dictated by the treating physician (surgeon or anaesthetist). No medication was given to patients without the approval and consent of either the study coordinator (ERAS group) or the treating physician (conventional care group). All patients were advised to lose 5–10 per cent of their preoperative bodyweight before surgery, as well as to cease smoking, alcohol and recreational drug use. If patients were on antidiabetic, pulmonary or cardiac medication, they underwent evaluation of their medication before surgery.

**Table 1:** Summary overview of protocols between study arms

	Conventional care	ERAS care
Before surgery	No counselling	Counselling about the contents and aims of the ERAS programme
	Premedication before surgery	No premedication before surgery
	No urinary catheter	No urinary catheter
Surgery	No infiltration of port sites	Infiltration of port sites with local anaesthetic
Anaesthesia	‘High propofol, low remifentanil’	‘Low propofol, high remifentanil’
	Invasive lines (such as blood pressure monitoring) on indication	No invasive lines
After surgery	Nasogastric tubing on indication	No nasogastric tubing
	No early oral feeding	Early oral feeding
	No early ambulation	Early ambulation
	Conventional administration of fluids	Restricted administration of fluids
	No clear antiemetic protocol, at discretion of caring staff	Clear antiemetic protocol
	No clear analgesic protocol, at discretion of caring staff	Clear analgesic protocol
	Opioids may be used routinely	Opioids may be used only as escape medication
Discharge	No predefined discharge date	Predefined discharge date

All LRYGB procedures were performed in a standard way with a 30–50‐ml gastric pouch, an antecolic/antegastric alimentary limb of 150 cm, a side‐to‐side 30‐mm linear stapled gastrojejunostomy, closed with an absorbable unidirectional barbed 3/0 V‐Loc™ suture (Covidien, Dublin, Ireland), and a side‐to‐side double‐stapled jejunojejunostomy with a 50‐cm biliary limb. The gastrojejunal anastomosis was tested for leakage with methylene blue dye through an orogastric tube, which was removed after the test. All surgeons used the same technique. No postoperative swallowing studies were done. A member of the study group was present during all operations, and assessed patients in recovery and on the surgical ward to ensure adherence to the protocol. A detailed overview of the study procedures, including medication dosages, is given in *Appendix* *S1* (supporting information).

Patients were discharged when all discharge criteria were met: pain adequately controlled (visual analogue scale score 4 or less) with paracetamol and non‐steroidal anti‐inflammatory drugs, fever and postoperative nausea and vomiting (PONV) absent, patient tolerating full liquid diet (sugar‐free custard pudding), able to mobilize independently, and feeling fit for discharge.

### Outcome measures

The primary endpoint was the functional hospital stay (FHS) in hours. This was the time from the end of the surgery until all discharge criteria had been met. Discharge criteria were assessed every hour, by either the study coordinators or ward nurses. Secondary endpoints were total hospital stay (THS) (time from surgery to discharge), 30‐day complication rate, death within 30 days, readmission within 30 days, duration of surgery and time spent on the recovery ward.

Thirty‐day complication rates were defined with the Clavien–Dindo classification for surgical complications^21^. Only complications of grade II or above (complications requiring pharmacological treatment with drugs other than antiemetics, antipyretics, analgesics, diuretics, electrolytes and physiotherapy) have been presented, as the inclusion of grade I complications (any deviation from the normal postoperative course) is of limited clinical value. Health‐related quality of life (HRQoL) was assessed with the Impact of Weight on Quality Of Life – Lite (IWQOL‐Lite©; Quality of Life Consulting, Durham, North Carolina, USA) and the Short Form 36 (SF‐36®; QualityMetric, Lincoln, Rhode Island, USA) questionnaires before and 30 days after bariatric surgery. Both questionnaires have been validated to measure HRQoL in a bariatric population^22^.

Patients were examined at the outpatient clinic 14 days after surgery and interviewed by telephone 30 days after surgery. During these contact moments, patients were asked about any complications. An electronic tag was added to each patient's chart, with a request to contact the study coordinator in case of an unscheduled visit to the emergency department or admission.

### Statistical analysis

In a sample of 203 patients who underwent LRYGB surgery in 2011, mean THS was 1·9 days (45·6 h). A decrease of 18 h was considered to be of clinical significance. Using a power calculator (nQuery Advisor for Windows® version 7.0; nQuery Advisor Software, Los Angeles, California, USA) with an α of 0·05 and a β of 0·80, the study was powered to detect this difference by recruiting 220 participants. This power calculation was performed on the secondary outcome (THS) instead of the primary outcome (FHS), because no data regarding FHS were available at the time.

Statistical analyses were performed using SPSS® for Windows® version 21 (IBM, Armonk, New York, USA). Continuous data are expressed as mean(s.d.) (parametric) or median (range) (non‐parametric). The Kolmogorov–Smirnov test was used to test for normality. Differences in continuous data between the two groups were analysed with either the independent *t* test (normally distributed data) or the Mann–Whitney *U* test (not normally distributed data). The χ^2^ test with Bonferroni correction was performed for categorical data. Two‐sided *P* < 0·050 was considered statistically significant. HRQoL analyses of within‐group differences (for example, before and after) were made with the paired‐samples *t* test or the Wilcoxon signed rank test, depending on the normality of the distribution. Between‐group differences at the endpoint (30 days after surgery) were calculated with the Mann–Whitney *U* test.

## Results

### Adherence to protocol

**Figure 1:**
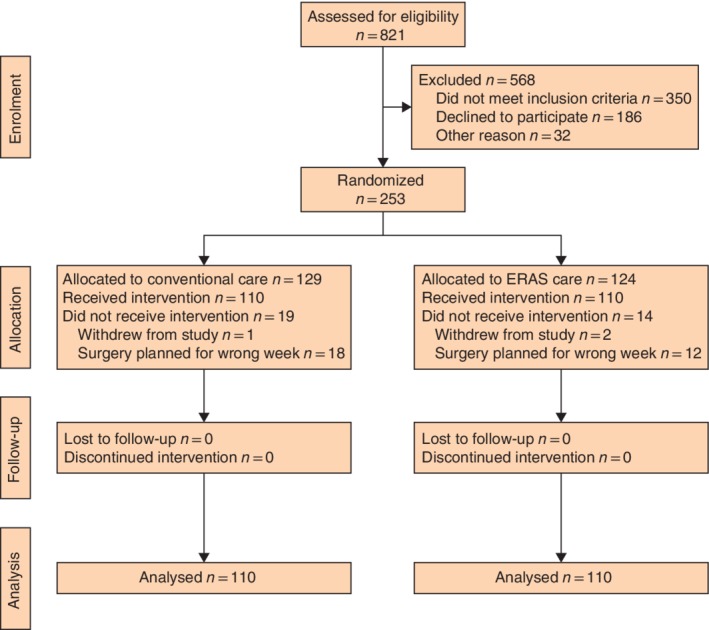
CONSORT diagram for the trial. ERAS, enhanced recovery after surgery

Of 821 patients assessed for eligibility, 568 were excluded (*Fig*. 1). A total of 253 patients scheduled for LRYGB were randomized between January 2013 and July 2014. Of these, 19 in the conventional care group and 14 in the ERAS group were excluded from analysis, either because they withdrew from participation before receiving the allocated treatment or because their surgery was planned for the wrong week. The baseline characteristics of these patients did not differ from those of patients who were included in the study. There were no other protocol violations.

### Patient characteristics

There were no baseline differences between the two study arms (*Table* 2). Mean age of the study participants was 42 years. Participants were predominantly women. Median preoperative BMI of the participants was 41·4 and 42·0 kg/m^2^ in conventional and ERAS arms respectively. Hypertension, dyslipidaemia and osteoarticular disease were the most common co‐morbidities in both groups.

**Table 2:** Baseline characteristics of included patients

	Conventional care (*n* = 110)	ERAS care (*n* = 110)
Age (years)*	42·6(10·8)	42·7(10·5)
Sex ratio (M : F)	16 : 94	12 : 98
Preoperative bodyweight (kg)†	118 (76·4–181·2)	121 (97·4–176)
Intraoperative BMI (kg/m^2^)†	41·4 (35–56)	42·0 (35·2–56·8)
Waist circumference (cm)†	121 (99–167)	123 (100–160)
OSMRS class		
A	79 (71·8)	76 (69·1)
B	30 (27·3)	34 (30·9)
C	1 (0·9)	0 (0)
Co‐morbidity		
T2DM	16 (14·5)	18 (16·4)
Hypertension	35 (31·8)	39 (35·5)
Dyslipidaemia	23 (20·9)	25 (22·7)
OSA	9 (8·2)	6 (5·5)
Osteoarticular	20 (18·2)	14 (12·7)
Cardiac	4 (3·6)	6 (5·5)
Pulmonary	19 (17·3)	14 (12·7)

Values in parentheses are percentages unless indicated otherwise; values are

* mean(s.d.) and

† median (range). ERAS, enhanced recovery after surgery; OSMRS, Obesity Surgery Mortality Risk Score; T2DM, type 2 diabetes mellitus; OSA, obstructive sleep apnoea.

### Primary outcome

FHS differed significantly between the study arms (median 20·5 h for conventional *versus* 17·4 h for ERAS care; *P* < 0·001) (*Table* 3).

**Table 3:** Hospital discharge times and time until individual discharge criteria were met

	Conventional care (*n* = 110)	ERAS care (*n* = 110)	*P* *
FHS (h)	20·5 (12·5–95·3)	17·4 (2·8–137·3)	< 0·001
THS (h)	21·2 (15·0–95·3)	21·3 (6·2–143·3)	0·343
Discharge criteria (h)			
VAS pain score ≤ 4	2·0 (0–52·5)	1·2 (0–25·0)	0·009
Fever	0·42 (0–21·1)	0·3 (0–18·0)	0·451
Full liquid diet tolerated	19·4 (0–45·7)	5·4 (0·9–66·3)	< 0·001
Nausea/vomiting	2·0 (0–44·6)	1·2 (0·2–22·9)	0·042
Mobilization	5·8 (0–27·2)	3·2 (0–32·3)	< 0·001
Tachycardia	0·5 (0–21·1)	0·3 (0–18·0)	0·814
Patient consent for discharge	20·0 (12·5–95·3)	17·2 (2·5–137·3)	< 0·001
Surgical time (min)	48·5 (30–165)	49 (20–120)	0·574
Time on recovery ward (h)	1·8 (1·0–4·5)	1·8 (0·8–3·6)	0·402

Values are median (range). ERAS, enhanced recovery after surgery; FHS, functional hospital stay; THS, total hospital stay; VAS, visual analogue scale.

* Mann–Whitney *U* test.

### Secondary outcomes

THS did not differ significantly between the study arms (21·5 h for conventional *versus* 21·3 h for ERAS care; *P* = 0·343). Pain control was achieved sooner in the ERAS group (by 1·2 h *versus* 2·0 h in the conventional group; *P* = 0·009). A full liquid diet was tolerated earlier in the ERAS group (after 5·4 h *versus* 19·4 h in the conventional group; *P* < 0·001). The time it took to control PONV was significantly lower in the ERAS group (1·2 *versus* 2·0 h respectively; *P* = 0·042). Patients in the ERAS group mobilized sooner than those who received conventional care (3·2 *versus* 5·8 hours after surgery; *P* < 0·001), and were comfortable with discharge about 3 h earlier (*P* < 0·001). No differences were found between the groups with respect to fever or tachycardia. Duration of surgery and time on recovery ward were not significantly different between the two groups (*Table* 3).

### Mortality, complications and readmission

No patient died. There were no differences in number of complications (Clavien–Dindo grade II or above), grade of complications or readmissions (*Table* 4). The most frequent complications were staple line bleeding, leakage, pneumonia, dehydration and trocar site infection.

**Table 4:** Thirty‐day complications and readmissions

	Conventional care (*n* = 110)	ERAS care (*n* = 110)	*P* *
Clavien–Dindo complication grade			
I	16 (14·5)	16 (14·5)	1·000
II	6 (5·5)	3 (2·7)	0·622
IIIa	1 (0·9)	2 (1·8)	1·000
IIIb	0 (0)	3 (2·7)	0·498
Any grade (≥ II)	7 (6·4)	8 (7·3)	1·000
Mortality	0 (0)	0 (0)	–
Readmission	3 (2·7)	7 (6·4)	0·333

Values in parentheses are percentages. ERAS, enhanced recovery after surgery.

* χ^2^ test.

### Health‐related quality of life

HRQoL data were not available for seven and ten patients before surgery, and for 27 and 25 patients at 30 days after surgery for conventional and ERAS groups respectively. SF‐36 ® and IWQOL‐Lite© general scores showed significant improvements after LRYGB surgery in both groups. There were no differences in HRQoL between the two groups (*Table* 5).

**Table 5:** Health‐related quality of life results

	Conventional care				ERAS care				Conventional *versus* ERAS care
	Before (*t* = 0) (*n* = 103)	After (*t* = 30) (*n* = 83)	*P*	Δ	Before (*t* = 0) (*n* = 100)	After (*t* = 30) (*n* = 85)	*P*	Δ	Δ *P*
SF‐36®									
Total score	68·8(15·7)	79·7(15·8)	< 0·001	−10·8(14·0)	71·2(14·9)	80·8(14·0)	< 0·001	−9·9(16·8)	0·267
Role limitations due to physical functioning	66·8(20·3)	84·1(20·2)	< 0·001	−17·1(20·2)	64·3(18·4)	83·8(18·0)	< 0·001	−19·6(20·6)	0·767
Role limitations due to physical health	70·4(37·4)	77·7(34·9)	0·153	−6·7(41·2)	78·3(36·5)	81·9(32·7)	0·580	−3·3(51·9)	0·250
Role limitations due to emotional problems	80·2(35·6)	88·8(30·5)	0·022	−9·5(35·8)	85·4(33·1)	89·6(28·0)	0·291	−5·0(40·3)	0·316
Energy/fatigue	57·0(18·9)	65·8(18·9)	< 0·001	−8·5(19·8)	62·7(16·3)	65·7(18·4)	0·099	−4·0(21·0)	0·058
Emotional well‐being	72·7(16·4)	80·7(16·4)	< 0·001	−8·0(12·8)	75·7(15·0)	82·7(14·1)	< 0·001	−7·5(16·4)	0·354
Social functioning	79·3(21·5)	83·3(23·0)	0·278	−2·9(23·4)	83·4(17·0)	83·8(20·3)	0·907	−0·3(24·4)	0·117
Pain	74·1(17·0)	80·7(23·0)	0·046	−5·0(21·5)	81·3(17·7)	86·0(18·8)	0·093	−4·5(23·5)	0·775
General health	66·0(17·3)	76·2(16·3)	< 0·001	−10·0(19·0)	65·8(16·8)	76·5(15·4)	< 0·001	−11·4(18·0)	0·710
IWQOL‐Lite©									
Total score	53·5(19·3)	82·8(19·7)	< 0·001	−30·1(21·8)	51·6(19·6)	81·3(21·5)	< 0·001	−30·3(30·0)	0·886
Physical function	46·1(23·6)	79·3(16·9)	< 0·001	−34·0(25·0)	43·1(22·5)	76·3(19·2)	< 0·001	−34·1(24·3)	0·863
Self‐esteem	46·6(26·9)	75·9(29·5)	< 0·001	−31·0(27·9)	45·7(26·9)	75·7(28·7)	< 0·001	−30·2(27·2)	0·244
Sex life	61·9(29·4)	75·4(30·5)	< 0·001	−14·6(32·3)	61·6(32·9)	78·1(28·2)	< 0·001	−17·7(27·5)	0·589
Public distress	60·5(27·0)	85·9(21·7)	< 0·001	−23·8(29·9)	56·0(28·7)	81·7(27·8)	< 0·001	−26·1(27·5)	0·628
Work	71·1(27·1)	88·8(21·4)	< 0·001	−16·8(27·4)	73·2(24·4)	90·0(20·4)	< 0·001	−17·4(19·7)	0·884

Values are mean(s.d.). ERAS, enhanced recovery after surgery; SF‐36®, Short Form 36 questionnaire; IWQOL, Impact of Weight on Quality Of Life questionnaire.

## Discussion

This study has shown that patients receiving ERAS care were ready for discharge 17·4 h after admission, and met discharge criteria with control of pain, no PONV, toleration of a full liquid meal and mobilization earlier. These outcomes were accomplished with no increase in postoperative morbidity or readmission rates.

These findings are in accordance with those of other studies. The only other RCT^19^ in bariatric surgery compared patients undergoing laparoscopic sleeve gastrectomy who received either ERAS or conventional care, and found that those in the ERAS group had a LOS of only 1 day, compared with 2 days for patients in the conventional care group. This decrease in LOS was not followed by an increase in readmission or perioperative morbidity rates^19^. Two meta‐analyses^23^, ^24^ have shown decreased LOS of 1·5 days with ERAS *versus* conventional care. None of these studies has, however, reported in detail how much each component of the ERAS programme could be attributed to the decrease in LOS. This makes it difficult to assess the added value of the decrease in time attributable to pain control, liquid diet tolerance, PONV or mobilization.

In the present study, the decrease in FHS in the ERAS group was 3 h, which was less than expected and less than that found in other studies^25^, ^26^. This outcome could be explained by the finding that patients in the conventional care group had an unexpectedly short FHS of 20·5 h. A possible explanation for this might be that a fast‐track programme was already in place at this centre from 2011^18^. Although the content of the present ERAS programme exceeded what was done in the fast‐track programme, it seems likely that the short FHS was influenced by the previously implemented fast‐track programme.

This study has several limitations. Thirty‐three randomized patients (19 in the conventional and 14 in the ERAS care group) were excluded from the analysis, mainly due to an error in the planning of operations (planned in the wrong week number). Their characteristics did not, however, differ from those of analysed patients. Like all other randomized trials investigating ERAS, this study was unblinded. The study coordinator and the caregivers all had to know the treatment arm in order to ensure adherence to the protocol. This might have resulted in some level of performance and detection bias. Although treatment arms were divided carefully, contamination of care cannot be excluded fully as the same ward staff tended to all patients. ERAS care contamination of the conventional care group might have contributed to the unexpectedly short FHS of this group. There were also few superobese patients in the sample, and revisional surgeries were excluded.

The FHS or LOS is the primary outcome in most studies investigating ERAS programmes because it is a sensitive marker for evaluation of their effectiveness. The disadvantage of using LOS as primary outcome measure is that it is often seen as a goal in itself. In the wake of an ever‐shortening LOS, there has been much debate about the safety of early discharge. Early discharge might lead to an increase in readmissions. The number of readmissions in the ERAS group in the present study was more than double that in the conventional care group, although this difference was not statistically significant. Morton and co‐workers^27^ used the Bariatric Outcomes Longitudinal Database (BOLD) to compare perioperative morbidity and mortality in patients with a 1‐day LOS with a reference standard of 2 days in patients undergoing LRYGB. They found a nearly significant value for an increase in 30‐day mortality, but no difference for 30‐day serious complications. Another large database^28^ found higher unadjusted mortality rates and higher overall morbidity for same‐day discharge compared with 1‐day discharge in patients having LRYGB. The results of these studies, however, have not been reproduced by large prospective series of LRYGB, nor are they confirmed by the present RCT. In the present study, the implementation of ERAS led to earlier recovery in comparison with conventional care, with no increase in 30‐day morbidity, and can therefore be recommended for patients undergoing LRYGB.

## Supporting information


**Appendix S1** Medication dosages

## References

[1] Sjöström L , Lindroos AK , Peltonen M , Torgerson J , Bouchard C , Carlsson B *et al.*; Swedish Obese Subjects Study Scientific Group . Lifestyle, diabetes, and cardiovascular risk factors 10 years after bariatric surgery. N Engl J Med 2004; 351: 2683–2693. 15616203 10.1056/NEJMoa035622

[2] Schauer PR , Bhatt DL , Kirwan JP , Wolski K , Brethauer SA , Navaneethan SD *et al.*; STAMPEDE Investigators . Bariatric surgery *versus* intensive medical therapy for diabetes – 3‐year outcomes. N Engl J Med 2014; 370: 2002–2013. 24679060 10.1056/NEJMoa1401329 PMC5451259

[3] Angrisani L , Santonicola A , Iovino P , Formisano G , Buchwald H , Scopinaro N . Bariatric surgery worldwide 2013. Obes Surg 2015; 25: 1822–1832. 25835983 10.1007/s11695-015-1657-z

[4] Schauer P , Ikramuddin S , Hamad G , Gourash W . The learning curve for laparoscopic Roux‐en‐Y gastric bypass is 100 cases. Surg Endosc 2003; 17: 212–215. 12457218 10.1007/s00464-002-8857-z

[5] Higa KD , Boone KB , Ho T . Complications of the laparoscopic Roux‐en‐Y gastric bypass: 1040 patients – what have we learned? Obes Surg 2000; 10: 509–513. 11175957 10.1381/096089200321593706

[6] Longitudinal Assessment of Bariatric Surgery (LABS) Consortium , Flum DR , Belle SH , King WC , Wahed AS , Berk P *et al.* Perioperative safety in the longitudinal assessment of bariatric surgery. N Engl J Med 2009; 361: 445–454. 19641201 10.1056/NEJMoa0901836 PMC2854565

[7] Maciejewski ML , Winegar DA , Farley JF , Wolfe BM , DeMaria EJ . Risk stratification of serious adverse events after gastric bypass in the Bariatric Outcomes Longitudinal Database. Surg Obes Relat Dis 2012; 8: 671–677. 23058451 10.1016/j.soard.2012.07.020

[8] Stenberg E , Szabo E , Agren G , Näslund E , Boman L , Bylund A *et al.*; Scandinavian Obesity Surgery Registry Study Group . Early complications after laparoscopic gastric bypass surgery: results from the Scandinavian Obesity Surgery Registry. Ann Surg 2014; 260: 1040–1047. 24374541 10.1097/SLA.0000000000000431

[9] Nguyen NT , Paya M , Stevens CM , Mavandadi S , Zainabadi K , Wilson SE . The relationship between hospital volume and outcome in bariatric surgery at academic medical centers. Ann Surg 2004; 240: 586–593. 15383786 10.1097/01.sla.0000140752.74893.24 PMC1356460

[10] Courcoulas A , Schuchert M , Gatti G , Luketich J . The relationship of surgeon and hospital volume to outcome after gastric bypass surgery in Pennsylvania: a 3‐year summary. Surgery 2003; 134: 613–621. 14605622 10.1016/s0039-6060(03)00306-4

[11] Flum DR , Salem L , Elrod JA , Dellinger EP , Cheadle A , Chan L . Early mortality among Medicare beneficiaries undergoing bariatric surgical procedures. JAMA 2005; 294: 1903–1908. 16234496 10.1001/jama.294.15.1903

[12] Kehlet H. Multimodal approach to control postoperative pathophysiology and rehabilitation. Br J Anaesth 1997; 78: 606–617. 9175983 10.1093/bja/78.5.606

[13] Carli F. Physiologic considerations of enhanced recovery after surgery (ERAS) programs: implications of the stress response. Can J Anaesth 2015; 62: 110–119. 25501695 10.1007/s12630-014-0264-0

[14] Kehlet H , Wilmore DW . Evidence‐based surgical care and the evolution of fast‐track surgery. Ann Surg 2008; 248: 189–198. 18650627 10.1097/SLA.0b013e31817f2c1a

[15] Kehlet H. Fast‐track surgery – an update on physiological care principles to enhance recovery. Langenbecks Arch Surg 2011; 396: 585–590. 21468643 10.1007/s00423-011-0790-y

[16] Wilmore DW , Kehlet H . Management of patients in fast track surgery. BMJ 2001; 322: 473–476. 11222424 10.1136/bmj.322.7284.473 PMC1119685

[17] Vlug MS , Wind J , Hollmann MW , Ubbink DT , Cense HA , Engel AF *et al.*; LAFA study group . Laparoscopy in combination with fast track multimodal management is the best perioperative strategy in patients undergoing colonic surgery: a randomized clinical trial (LAFA‐study). Ann Surg 2011; 254: 868–875. 21597360 10.1097/SLA.0b013e31821fd1ce

[18] Geubbels N , Bruin SC , Acherman YI , van de Laar AW , Hoen MB , de Brauw LM . Fast track care for gastric bypass patients decreases length of stay without increasing complications in an unselected patient cohort. Obes Surg 2014; 24: 390–396. 24254930 10.1007/s11695-013-1133-6

[19] Lemanu DP , Singh PP , Berridge K , Burr M , Birch C , Babor R *et al.* Randomized clinical trial of enhanced recovery *versus* standard care after laparoscopic sleeve gastrectomy. Br J Surg 2013; 100: 482–489. 23339040 10.1002/bjs.9026

[20] Thorell A , MacCormick AD , Awad S , Reynolds N , Roulin D , Demartines N *et al.* Guidelines for perioperative care in bariatric surgery: Enhanced Recovery After Surgery (ERAS) Society recommendations. World J Surg 2016; 40: 2065–2083. 26943657 10.1007/s00268-016-3492-3

[21] Clavien PA , Barkun J , de Oliveira ML , Vauthey JN , Dindo D , Schulick RD *et al.* The Clavien–Dindo classification of surgical complications: five‐year experience. Ann Surg 2009; 250: 187–196. 19638912 10.1097/SLA.0b013e3181b13ca2

[22] De Zwaan M , Mitchell JE , Howell LM , Monson N , Swan‐Kremeier L , Roerig JL *et al.* Two measures of health‐related quality of life in morbid obesity. Obes Res 2002; 10: 1143–1151. 12429878 10.1038/oby.2002.155

[23] Singh PM , Panwar R , Borle A , Goudra B , Trikha A , van Wagensveld BA *et al.* Efficiency and safety effects of applying ERAS protocols to bariatric surgery: a systematic review with meta‐analysis and trial sequential analysis of evidence. Obes Surg 2017; 27: 489–501. 27878754 10.1007/s11695-016-2442-3

[24] Ahmed OS , Rogers AC , Bolger JC , Mastrosimone A , Robb WB . Meta‐analysis of enhanced recovery protocols in bariatric surgery. J Gastrointest Surg 2018; 22: 964–972. 29488124 10.1007/s11605-018-3709-x

[25] Dogan K , Kraaij L , Aarts EO , Koehestanie P , Hammink E , van Laarhoven CJ *et al.* Fast‐track bariatric surgery improves perioperative care and logistics compared to conventional care. Obes Surg 2015; 25: 28–35. 24993524 10.1007/s11695-014-1355-2

[26] Mannaerts GH , van Mil SR , Stepaniak PS , Dunkelgrün M , de Quelerij M , Verbrugge SJ *et al.* Results of implementing an enhanced recovery after bariatric surgery (ERABS) protocol. Obes Surg 2016; 26: 303–312. 26003552 10.1007/s11695-015-1742-3

[27] Morton JM , Winegar D , Blackstone R , Wolfe B . Is ambulatory laparoscopic Roux‐en‐Y gastric bypass associated with higher adverse events? Ann Surg 2014; 259: 286–292. 24169190 10.1097/SLA.0000000000000227

[28] Inaba CS , Koh CY , Sujatha‐Bhaskar S , Zhang L , Nguyen NT . Same‐day discharge after laparoscopic Roux‐en‐Y gastric bypass: an analysis of the metabolic and bariatric surgery accreditation and quality improvement program database. J Am Coll Surg 2018; 226: 868–873. 29428234 10.1016/j.jamcollsurg.2018.01.049 PMC6152887

