# Supplementary material for: Randomized clinical trial of an enhanced recovery after surgery programme versus conventional care in laparoscopic Roux‐en‐Y gastric bypass surgery
Source: BJS Open. 2019 Mar 18;3(3):274–81. doi: 10.1002/bjs5.50143 (PMC6551390; doi:10.1002/bjs5.50143)
Supplement: Supplementary file 1 — Appendix S1 Medication dosages [file BJS5-3-274-s001.docx]

**BJS5_50143**

**Randomized clinical trial of an enhanced recovery after surgery programme *versus* conventional care in laparoscopic Roux-en-Y gastric bypass surgery**

**N. Geubbels, I. Evren, Y. I. Z. Acherman, S. C. Bruin, A. W. J. M. van de Laar, M. B. Hoen and L. Maurits de Brauw**

**Appendix S1 Medication dosages**

Premedication:

| **Conventional care** | **ERAS care** |
| --- | --- |
| Oxazepam 10mg 1dd1, orally | Diclofenac 75mg 1dd1, orally |
| Diclofenac 75mg 1dd1, orally | Paracetamol 500mg 1dd2, orally |
| Paracetamol 500mg 1dd2, orally |  |

Anaesthetic protocol:

| **Conventional care, all based on IBW*** | **ERAS care, all based on IBW*** |
| --- | --- |
| **Induction:**  Target controlled infusion:  Propofol 1% 1-2,5mg/kg iv bolus  Remifentanil 0,5-1µg/kg/min  Rocuronium 0,6-1,2 mg/kg | **Induction:**  Bolus infusion :  Propofol 1% 2,5mg/kg  Sufentanil 0,25 µg/kg  Rocuronium 0,6-1,2 mg/kg  1 L 0,9% NaCl fluid therapy on induction |
| **Maintenance:**  Propofol 1% 50-200 µg/kg/min  Remifentanil 0,25 µg/kg/min | **Maintenance:**  Propofol 1% 72,9 – 93,8µg/kg/min  Remifentanil 0,75 – 1,125µg/kg/min |
| **Reversion of neuromuscular blockade:**  Sugammadex 2 – 16 mg/kg, dependent on depth of block  TOF > 90% = reversion | **Reversion of neuromuscular blockade:**  Sugammadex 2 – 16 mg/kg dependent on depth of block  TOF > 90% = reversion |
| **Antibiotic prophylaxis:**  Cefazoline 1gram, metronidazole 500mg bolus at induction | **Antibiotic prophylaxis:**  Cefazoline 1gram, metronidazole 500mg bolus at induction |
| **Thromboprophylaxis:**  Clexane 40mg 2dd1 subcutaneous, first dose 2 hours pre operatively.  Continuous therapy until 2 weeks postoperatively | **Thromboprophylaxis:**  Clexane 40mg 2dd1 subcutaneous, first dose 2 hours pre operatively.  Continuous therapy until 2 weeks postoperatively |

Antalgic protocol:

| **Conventional care** | **ERAS care** |
| --- | --- |
| In operating theatre:  Piritramide 5-10mg iv 20 min before end of surgery | No analgesics in operating theatre. |
| On recovery ward:  Entirely according to discretion of attending anaesthesiologist  Escape: entirely according to discretion of attending anaesthesiologist | On Recovery ward:  Piritramide 5mg iv (repeat op to 3 times, maximum dosage 20mg), until VAS ≤4  Escape on recovery:  Clonidine 75 µg iv. (repeat op to 1 time, maximum dosage 150 µg) |
| Surgical ward protocol:  Paracetamol 1000 mg 4dd1 , i.v. or orally Diclofenac 50mg 3dd1, orally  Escape: entirely according to the discretion of the surgical attending | Surgical ward protocol:  Ward protocol:  Paracetamol 1000 mg 4dd1  Diclofenac 50mg 3dd1, orally (up to 48 hours post surgery)  Escape on ward:  When patient indicates pain or VAS >4:  Piritramide 5 mg iv, thereafter 2,5 mg iv, untill VAS ≤4. |

Anti-emetic protocol:

| **Conventional care** | **ERAS care** |
| --- | --- |
| In operating theatre:  Entirely according to discretion of attending anaesthesiologist | In operating theatre:  Dexamethason 4mg iv bolus before induction  Granisetron 1mg 1iv bolus at end of surgery |
| Recovery/ward:  Entirely according to discretion of attending anaesthesiologist or surgical attending | Recovery/ward:  Granisetron 1mg iv (repeat up to 1 time, maximum dosage 3mg in 24 hours)  Escape:  Metoclopramide 20mg iv (repeat up to 1 time on patient demand, maximum dosage 40mg in 24 hours) |
